# Predicting treatment retention in medication for opioid use disorder: a machine learning approach using NLP and LLM-derived clinical features

**DOI:** 10.1093/jamia/ocaf157

**Published:** 2025-09-22

**Authors:** Fateme Nateghi Haredasht, Ivan Lopez, Steven Tate, Pooya Ashtari, Min Min Chan, Deepali Kulkarni, Chwen-Yuen Angie Chen, Maithri Vangala, Kira Griffith, Bryan Bunning, Adam S Miner, Tina Hernandez-Boussard, Keith Humphreys, Anna Lembke, L Alexander Vance, Jonathan H Chen

**Affiliations:** Stanford Center for Biomedical Informatics Research, Stanford, CA 94305, United States; Stanford University School of Medicine, Stanford, CA 94305, United States; Department of Biomedical Data Science, Stanford, CA 94305, United States; Department of Psychiatry & Behavioral Sciences, Stanford University School of Medicine, Stanford, CA 94305, United States; Department of Electrical Engineering (ESAT), STADIUS Center, KU Leuven, 3001 Leuven, Belgium; KKT Technologies, Pte. Ltd., 139951, Singapore; Holmusk Technologies, Inc., NY 10012, United States; KKT Technologies, Pte. Ltd., 139951, Singapore; Holmusk Technologies, Inc., NY 10012, United States; Division of Primary Care and Population Health, Department of Medicine, Stanford University School of Medicine, Stanford, CA 94305, United States; Holmusk Technologies, Inc., NY 10012, United States; Holmusk Europe, Ltd., London, United Kingdom; Department of Biomedical Data Science, Stanford, CA 94305, United States; Department of Psychiatry & Behavioral Sciences, Stanford University School of Medicine, Stanford, CA 94305, United States; Stanford Center for Biomedical Informatics Research, Stanford, CA 94305, United States; Department of Psychiatry & Behavioral Sciences, Stanford University School of Medicine, Stanford, CA 94305, United States; Veterans Affairs Health Care System, Palo Alto, CA 94304, United States; Department of Psychiatry & Behavioral Sciences, Stanford University School of Medicine, Stanford, CA 94305, United States; Holmusk Technologies, Inc., NY 10012, United States; Stanford Center for Biomedical Informatics Research, Stanford, CA 94305, United States; Division of Hospital Medicine, Stanford University School of Medicine, Stanford, CA 94305, United States; Clinical Excellence Research Center, Stanford School of Medicine, Stanford, CA 94305, United States; Department of Medicine, Stanford, CA 94305, United States

**Keywords:** opioid use disorder, treatment attrition, machine learning, natural language processing, large language models, electronic health records, predictive modeling

## Abstract

**Objective:**

Building upon our previous work on predicting treatment retention in medications for opioid use disorder, we aimed to improve 6‐month retention prediction in buprenorphine-naloxone (BUP-NAL) therapy by incorporating features derived from large language models (LLMs) applied to unstructured clinical notes.

**Materials and Methods:**

We used de-identified electronic health record (EHR) data from Stanford Health Care (STARR) for model development and internal validation, and the NeuroBlu behavioral health database for external validation. Structured features were supplemented with 13 clinical and psychosocial features extracted from free-text notes using the CLinical Entity Augmented Retrieval pipeline, which combines named entity recognition with LLM-based classification to provide contextual interpretation. We trained classification (Logistic Regression, Random Forest, XGBoost) and survival models (CoxPH, Random Survival Forest, Survival XGBoost), evaluated using Receiver Operating Characteristic-Area Under the Curve (ROC-AUC) and *C*-index.

**Results:**

XGBoost achieved the highest classification performance (ROC-AUC = 0.65). Incorporating LLM-derived features improved model performance across all architectures, with the largest gains observed in simpler models such as Logistic Regression. In time-to-event analysis, Random Survival Forest and Survival XGBoost reached the highest *C*-index (≈0.65). SHapley Additive exPlanations analysis identified LLM-extracted features like Chronic Pain, Liver Disease, and Major Depression as key predictors. We also developed an interactive web tool for real-time clinical use.

**Discussion:**

Features extracted using NLP and LLM-assisted methods improved model accuracy and interpretability, revealing valuable psychosocial risks not captured in structured EHRs.

**Conclusion:**

Combining structured EHR data with LLM-extracted features moderately improves BUP-NAL retention prediction, enabling personalized risk stratification and advancing AI-driven care for substance use disorders.

## Background

The opioid epidemic remains a significant public health crisis, with opioid use disorder (OUD) affecting millions of individuals worldwide and non-medical opioid use linked to a tenfold increase in mortality.^1^ Medications for opioid use disorder (MOUD), such as buprenorphine-naloxone (BUP-NAL), have demonstrated efficacy in reducing opioid-related morbidity and mortality. However, a major challenge in OUD treatment is retention, as patients often discontinue MOUD prematurely. Retention rates for buprenorphine treatment range widely from 20% to 82.5%,^2^ with many patients discontinuing treatment within the first 6 months.^3^ Among oral forms of MOUD in the United States, buprenorphine can have more flexible prescribing models than methadone (which is generally available only through federally certified opioid treatment programs), but buprenorphine demonstrates lower retention rates than methadone.^4–6^ Early discontinuation of buprenorphine therapy is associated with increased mortality, underscoring the need for identifying patients at risk for treatment attrition for targeted interventions and health system assessments.^4^

Predictive modeling is a powerful tool for identifying patients at risk of discontinuing MOUD.^7–16^ Traditional risk prediction models rely heavily on structured electronic health record (EHR) data.^17–19^ Structured data includes discrete, coded clinical variables that are systematically recorded in the EHR, including patient demographics (eg, age, sex), medical diagnoses (eg, ICD-10 codes), prescribed medications (eg, RxNorm codes), laboratory test results, and procedural records (eg, CPT codes). These features are stored in predefined formats, which are convenient for computational analysis.

Although these structured data elements provide important insights, they cannot capture the complete clinical context. Unstructured clinical data includes notes within EHRs written in free-text (no explicitly defined structure) that can capture richer contextual information.^20^^,^^21^ These free-text notes—containing physician observations, patient-reported concerns, and social determinants of health—can offer unique qualitative insights into factors that are more often not captured in structured diagnosis codes, such as psychiatric comorbidities, psychosocial stressors, substance use history, and overall patient engagement, all of which influence MOUD treatment retention.^22^^,^^23^ Utilizing this information for large-scale analyses is challenging due to the complexity and variability of unstructured clinical note data, necessitating advanced natural language processing (NLP) techniques to organize and extract relevant information out of unstructured EHR data. Our prior work suggested NLP methods using keyword identification techniques could modestly improve MOUD treatment retention prediction models, but were limited by their dependence on predefined keywords and their inability to grasp context.^19^ For example, prior methods could look for keywords like “homelessness” but would completely miss a case where the documented note describes a patient “living in his car,” given no overlap in wording. The advent of large language models (LLMs) has enabled more sophisticated methods for extracting clinically relevant information from unstructured text with more “reasoning” around the context and semantics of identified clinical concepts.^24–26^

This study leverages our NLP pipeline called CLinical Entity Augmented Retrieval (CLEAR), which combines LLMs and information retrieval methods to extract interpretable clinical features relevant to MOUD from clinical notes often missed by structured data approaches.^27^^,^^28^ In this study, we assess whether key psychosocial risk factors for MOUD treatment attrition can be extracted using these advanced LLM and NLP techniques and whether this improves treatment retention prediction, accessible through an interactive web risk calculator.

## Methods

### Study design and data sources

This study utilized de-identified datasets to train and validate the predictive models that predict BUP-NAL treatment retention vs attrition, with external validation performed using data from separate patient cohorts. The study population consisted of patients aged 16-89 years who had received at least one prescription for BUP-NAL for more than 1 day. Treatment duration was defined as the period between the start and end of consecutive BUP-NAL prescriptions. Continuous treatment was considered to be the case if the gap between the end date of one prescription and the start date of the next did not exceed 30 days. Treatment attrition was defined as a treatment duration of fewer than 180 days, consistent with prior research and established quality-of-care benchmarks.^29^^,^^30^ The Stanford EHR dataset was used for model development and internal validation, and NeuroBlu, a longitudinal behavioral health database, was used for external validation to assess model generalizability across different healthcare settings. All data were de-identified following the Safe Harbor method in accordance with the National Institute of Standards and Technology guidelines, and clinical text was further anonymized using the TiDE algorithm.^31^ This study was approved by the Stanford University Institutional Review Board (IRB #67423).

### Stanford electronic health record data (STARR)

The STAnford Research Repository (STARR) dataset is an integrated health system database that includes de-identified EHR data from an academic hospital (Stanford Health Care), a community hospital (ValleyCare Hospital), and a community practice network (University Healthcare Alliance). It encompasses both inpatient and outpatient encounters across various specialties from 1999 to 2022. To enable comparisons across institutions, the STARR data were standardized to the Observational Medical Outcomes Partnership (OMOP) Common Data Model (CDM).^32^ The subset of the STARR dataset utilized for this study included 1800 treatment encounters for 1272 unique patients receiving BUP-NAL prescriptions, along with 13 922 unique clinical notes. Of these encounters, 1099 (61%) resulted in treatment attrition within 6 months of initiating BUP-NAL. In the Stanford dataset, treatment start dates up to 2020 were used for model training, and treatment start dates from 2021 onwards were used for validation.

### NeuroBlu behavioral health database

NeuroBlu is a real-world behavioral health database that aggregates de-identified EHR data from all 50 US states from 166 distinct sites of care, including hospitals, emergency rooms, and community psychiatry clinics spanning over 20 years (2003-2024).^33^ The portion of the dataset utilized for this study was standardized to the OMOP CDM format. After standardizing the data and selecting patients who met the inclusion criteria with clinical note data available, the final dataset for analysis included 459 treatment encounters for 342 unique patients, with 11 681 clinical notes. Among these encounters, 381 (83%) were classified as attrition within 6 months of BUP-NAL initiation.

### Feature extraction

Feature extraction involved 2 main phases: the extraction of baseline structured EHR data and additional features derived from unstructured notes using the CLEAR NLP pipeline described below.^27^

### Structured data

**Figure 1.**
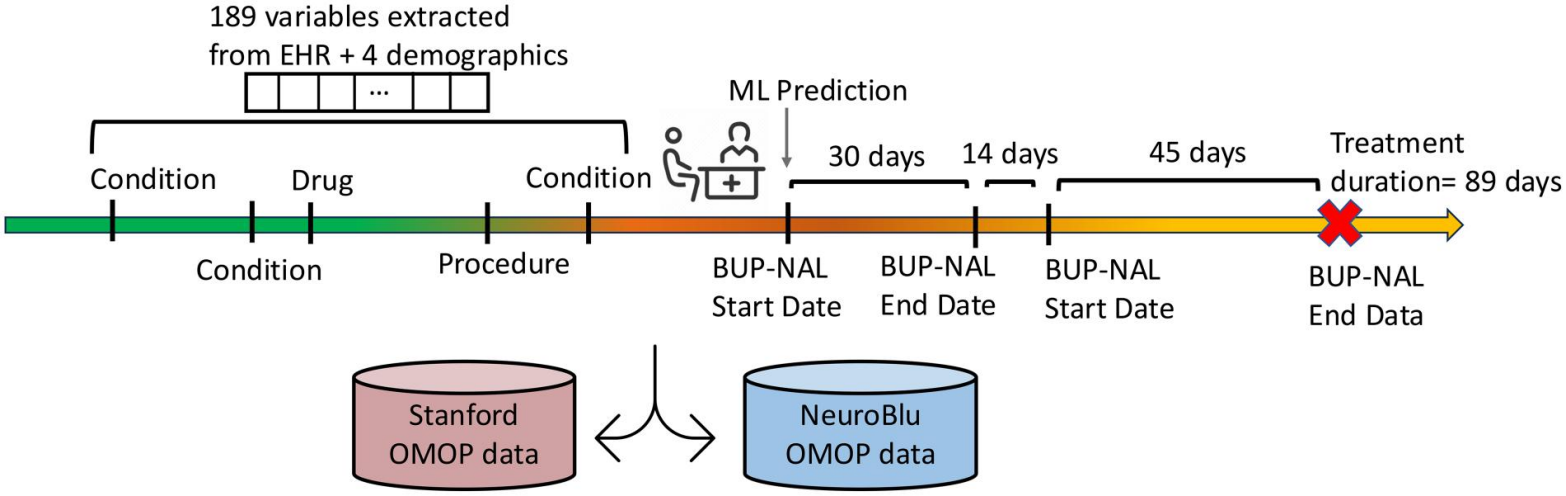
Timeline representation of a patient with feature extraction and treatment tracking for BUP-NAL therapy. A common set of 189 features, including diagnoses, medications, and procedural records, plus 4 demographic features, is extracted from patient data in the Stanford and NeuroBlu OMOP datasets. The prediction index time is set at the onset of a BUP-NAL treatment period when continuous prescriptions persist without a treatment gap of more than 30 days. The treatment duration is illustrated up to the red “X” after a continuous period of 89 days in this example.

The datasets contain 17 961 diagnostic markers, 17 271 procedural indicators, and 47 476 drug-associated features. To select features significantly associated with treatment retention, both sites independently applied association rule mining with Fisher’s exact test (*P* < .05) to each dataset. This resulted in the structured data from the Stanford and NeuroBlu datasets, including 578 and 636 candidate features, respectively, covering domains including demographics, diagnoses, medications, and procedures. We created a common standardized feature dictionary by mapping the overlapping features across both datasets, resulting in 189 shared features plus 4 demographic features used for model development. Figure 1 illustrates the processing of structured EHR data from both datasets, and a complete list of the structured features is provided in Table S1.

Patient age was treated as a continuous variable. All other features were encoded as binary indicators (1 = present, 0 = absent), naturally addressing missing values without the need for imputation.

### Unstructured data from clinical notes

A board-certified addiction medicine physician, S.T., reviewed previous literature to identify 13 key features related to treatment attrition from unstructured clinical notes relevant in clinical practice.^20^ These features included indications of post-traumatic stress disorder, major depression, homelessness, personality disorders, tobacco dependence, bipolar disorder, attention deficit hyperactivity disorder, substance use disorders excluding OUD, chronic pain, suicidal behavior, unemployment, alcohol dependence, and liver disease. These features were extracted from the clinical notes using the CLEAR pipeline, which combines named entity recognition methods with LLMs to extract information as a form of Retrieval-Augmented Generation (RAG)^28^ described below. While LLMs enhance the interpretability of extracted mentions, the CLEAR pipeline does not represent a fully generative or end-to-end LLM-based system.

CLEAR requires 2 inputs: clinical notes and a target entity (eg, “homelessness”). First, it uses a named-entity recognition model (Flan-T5-XXL in this case) to scan notes and extract all clinical entities mentioned (eg, “depression,” “colorectal cancer,” and “homelessness”). Next, the extracted entities are filtered to the ones of interest and then augmented with synonyms and morphological variations from medical ontologies (eg, UMLS) and LLM-based paraphrasing (using GPT-4). This augmentation process allows the process to capture wording variations. For example, the process can find wordings relevant to homelessness (eg, “unhoused” or “unstable housing”), even when there is no direct overlap in words or phrases. Following this, the system retrieves segments of notes by extracting text surrounding each mention of the target entity or its associated terms (“homelessness,” “unhoused,” “unstable housing”). Finally, these note segments are provided to an LLM with a tailored prompt (eg, “Is this patient experiencing homelessness?”) to classify the presence or absence of the target condition.

A detailed depiction of the CLEAR pipeline workflow can be found in Figure S1. Output from the CLEAR process produced binary labels (present vs absent) for each of the 13 features identified by our clinical expert. To ensure the accuracy of the extracted features, the pipeline underwent human validation, where clinical experts assessed the correctness of LLM-derived assertions. The agreement between the CLEAR pipeline and human annotations exceeded 90% on average, as measured by the F1 score (see Supplementary S2 and Lopez et al.^27^ for additional details). As shown in Table S2, GPT-4 consistently outperformed other LLMs (including Med42, Llama-3, and Flan-UL2), achieving the highest overall average F1 score of 0.97.

**Figure 2.**
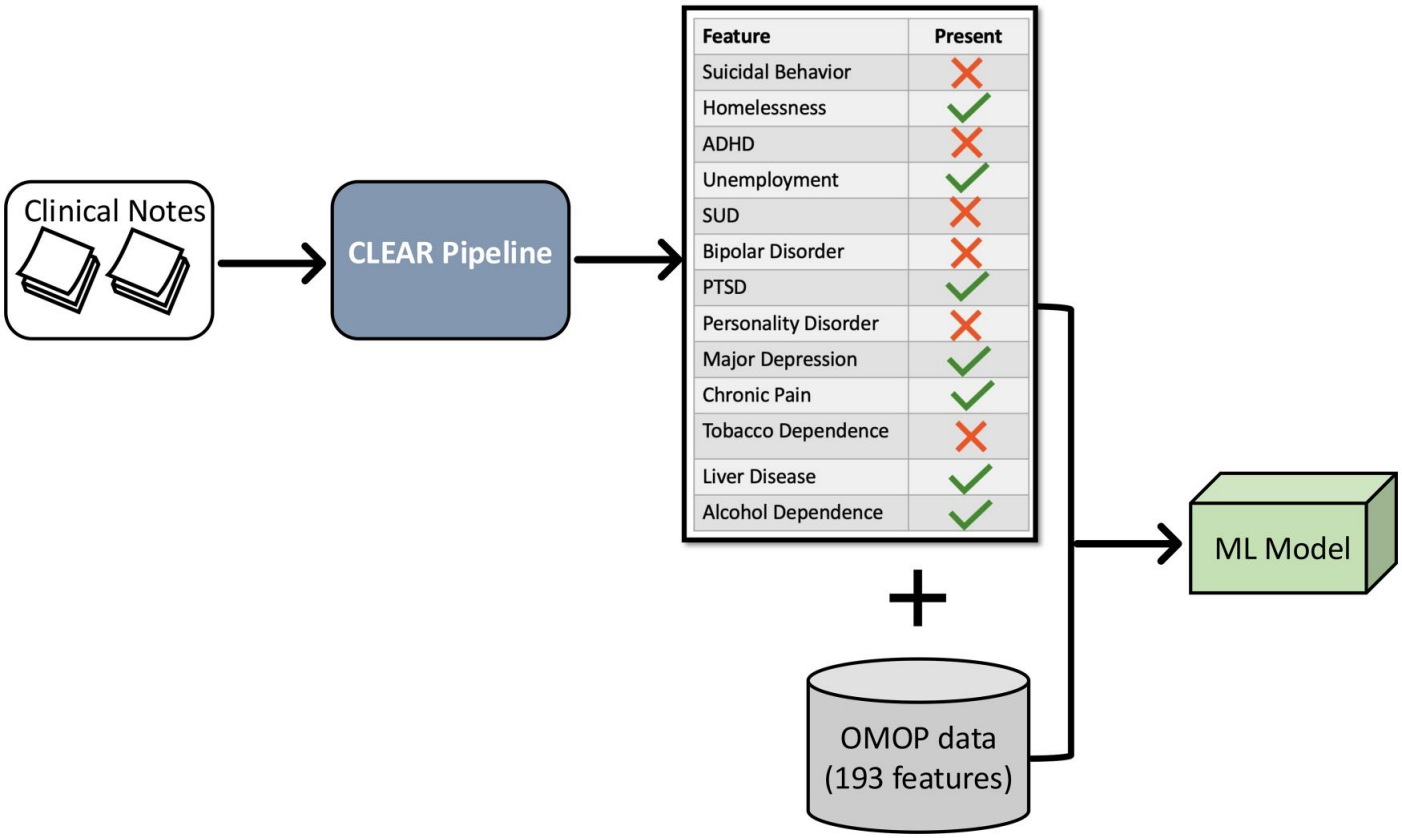
Data processing workflow for feature extraction and model training. Clinical notes are processed through the CLEAR pipeline, which extracts relevant features using LLMs. The output from CLEAR is combined with structured EHR data. This resulted in 206 features in common across all study sites and was used to train and validate the machine learning models.

Only features documented from clinical notes recorded at least 1 day before the index BUP-NAL prescription date were used, and if a feature appeared multiple times across different notes, it was counted once. The workflow for unstructured feature extraction and integration is illustrated in Figure 2.

### Model development

We developed machine learning models using 2 main modeling approaches: classification to predict attrition or retention at the 6-month mark and time-to-event analysis to model dynamic treatment.

#### Classification for attrition prediction

We trained models on Stanford data using 206 features (193 from structured data, including demographics, and 13 from unstructured data) to predict whether a patient would experience treatment attrition within 6 months of starting BUP-NAL therapy. The evaluated classification models include Logistic Regression, Random Forest,^34^ and XGBoost^35^ classifiers for binary prediction of treatment attrition vs retention. These methods were chosen for their demonstrated effectiveness in prior research predicting attrition in OUD treatment.^36^

Each model underwent hyperparameter tuning using grid search with cross-validation on the Stanford dataset to optimize performance. The models’ performance was internally validated using the Stanford dataset and externally validated using the NeuroBlu dataset.

To assess model performance, we used precision, recall, F1-score, and Receiver Operating Characteristic—Area Under the Curve (ROC-AUC) as standard evaluation metrics. Precision (positive predictive value) measures the proportion of predicted attrition cases that were correctly identified, where a higher precision indicates fewer false positives. Recall (sensitivity) evaluates the proportion of actual attrition cases that the model correctly identified, with higher recall reflecting fewer false negatives. F1-score is the harmonic mean of precision and recall, providing a balanced measure of model performance when false positives and false negatives carry similar importance. Lastly, ROC-AUC quantifies the model’s ability to discriminate between retention and attrition cases by summarizing the tradeoff between sensitivity and specificity. A higher ROC-AUC score, approaching 1.0, indicates perfect discrimination and ranking of high vs low risk cases, whereas an ROC-AUC of 0.5 indicates risk predictions no better than random guessing.

The robustness of model performance was further assessed through 50 iterations of testing on resampled test data, with average values and standard deviations reported. Our approach to developing and evaluating machine learning models adheres to established practices,^37^ and the design and reporting of this study conform to the TRIPOD reporting guidelines for risk prediction models.^38^^,^^39^

### Time-to-event analysis

In addition to binary prediction of treatment attrition, we conducted a time-to-event survival analysis to focus on understanding the duration of treatment retention, that is, how long a patient stays in treatment, estimating the probability of discontinuation at different times while accounting for censored data. This analysis provided insights into the duration of BUP-NAL therapy by allowing us to model the likelihood and timing of attrition.

For this purpose, we employed Cox proportional hazards (CoxPH),^40^ Random Survival Forest (RSF),^41^ and Survival XGBoost^35^ models. Feature selection and the number of features to use for these models were identical to those used for the binary classifier models. Models were trained and internally validated on the Stanford dataset and externally validated on the NeuroBlu dataset.

We used the concordance index (*C*-index)^42^ to measure the models’ ability to rank survival times accurately, analogous to the ROC-AUC for binary classification. A higher *C*-index indicates a better ranking of patients by their treatment duration, with a *C*-index of 1.0 signifying a perfect ranking.

Each model underwent 5-fold cross-validation, repeated 10 times, totaling 50 evaluations per model. We calculated the average *C*-index and standard deviations to assess performance and consistency. The scikit-survival package was used for these analyses.^43^

### Feature importance and sensitivity analysis

We used SHapley Additive exPlanations (SHAP)^44^ to quantify the top 15 most important features influencing model predictions.

To evaluate the added predictive value of features extracted from unstructured data, we conducted a sensitivity analysis using Stanford data. We compared model performance (ROC-AUC for classification and *C*-index for time-to-event) across 2 configurations: (1) a model with 193 features from structured data only and (2) a model with a full 206 features, including 13 features from unstructured data. To assess the statistical significance of performance differences, we applied 3 statistical tests—Wilcoxon signed-rank, Mann–Whitney U, and paired t-tests—for each model comparison.

To further address redundancy and explore the interplay between structured and unstructured features, we compared 4 model configurations: a structured-only model, an NLP-only model using just the 13 LLM-derived features, a combined model appending all features without harmonization, and a harmonized model where overlapping features were reconciled into unified binary indicators (eg, “depression from any source”). All models were trained using Random Survival Forests with 5-fold cross-validation repeated over 100 runs and evaluated using the *C*-index.

Finally, we conducted a subgroup analysis stratified by age, gender, race, and ethnicity. For each subgroup, model performance was assessed using the *C*-index from 5-fold cross-validation RSF.

### Web application for interactive use

To enhance the translation of our predictive model into clinical workflows, we created an interactive web application that enables users to visualize predictions in real time. We utilized the top 15 important features extracted from SHAP to develop and deploy this model. The application allows clinicians and researchers to enter individual patient clinical variables interactively to dynamically generate a custom risk prediction report and personalized “survival” risk curve.

## Results

### Feature prevalence

Table 1 provides the demographic characteristics of the Stanford and NeuroBlu datasets. Individual patients may have multiple encounters if they initiated BUP-NAL treatment more than once after a treatment gap (more than 30 days). In the Stanford dataset, 28% of patients experienced multiple encounters, and in the NeuroBlu dataset, this percentage was 34%.

**Table 1.** The demographic characteristics of the Stanford and NeuroBlu datasets, which represent encounters rather than unique patients, as individuals may have multiple encounters of care within the study period.

Feature	Stanford dataset	NeuroBlu dataset
Age (years)		
Mean (SD)	49.4 (16.5)	42.2 (12.4)
Median (IQR)	51 (28)	40 (18)
Sex		
Male	52.8%	59.3%
Female	47.2 %	40.7%
Race		
White	72.1%	43.4%
Black or African American	6.6%	55.6%
Other Race	5.1%	0.4%
Not reported	16.2%	0.7%
Ethnicity		
Hispanic or Latino	12.4%	0%
Not Hispanic or Latino	84.5%	0.4%
Not reported	3.1%	99.6%

The 2 datasets show notable differences in their demographics. The Stanford dataset has a higher mean age (49.4 years) compared to the NeuroBlu dataset (42.2 years). Most patients in the Stanford dataset were identified as White (72.1%), whereas the NeuroBlu dataset has a majority of Black or African American individuals (55.6%). Additionally, 12.4% of Stanford participants are Hispanic or Latino, whereas none in NeuroBlu are.

Additionally, Table S3 reports the prevalence of 13 LLM-derived features across the 2 datasets based on the number of encounters. Patients in the NeuroBlu dataset showed higher rates of psychiatric comorbidities, particularly bipolar disorder and depression, compared to the Stanford cohort.

A correlation heatmap (Figure S2) visualizes the relationships among the 13 features extracted from unstructured data using Stanford data. Strong positive correlations were observed between Chronic Pain and Substance Use Disorder, as well as between Alcohol Dependence and Substance Use Disorder.

### Model performance

The performance of both classification and time-to-event models is summarized in Tables 2 and 3. In Table 2, which presents results for classification models, Random Forest achieved the highest recall across both datasets, while XGBoost achieved the highest precision. XGBoost had the best overall ROC-AUC on the Stanford data. Logistic Regression had the lowest performance across all metrics but improved notably when LLM-derived features were included (detailed in the Sensitivity Analysis section).

**Table 2.** Classification performance of machine learning models trained on Stanford EHR data in terms of predicting whether a patient starting BUP-NAL therapy will stop treatment within 180 days.

Source	Model	Precision	Recall	F1-score	ROC-AUC
Stanford (internal validation)	Logistic Regression	0.69 ± 0.02	0.52 ± 0.03	0.57 ± 0.03	0.57 ± 0.03
	Random Forest	0.70 ± 0.01	0.77 ± 0.02	0.72 ± 0.02	0.64 ± 0.03
	XGBoost	0.72 ± 0.02	0.75 ± 0.02	0.71 ± 0.02	0.65 ± 0.03
NeuroBlu (external validation)	Logistic Regression	0.90 ± 1.00	0.20 ± 0.07	0.32 ± 0.03	0.60 ± 0.03
	Random Forest	0.83 ± 0.01	0.78 ± 0.02	0.69 ± 0.02	0.56 ± 0.03
	XGBoost	0.86 ± 0.01	0.56 ± 0.03	0.64 ± 0.07	0.57 ± 0.03

Precision (positive predictive value), recall (sensitivity), F1-score, and ROC-AUC are reported for the Logistic Regression, Random Forest, and XGBoost models.

**Table 3.** Time-to-event analysis performance of survival models across the Stanford and NeuroBlu datasets.

Source	Model	*C*-index
Stanford (internal validation)	CoxPH	0.63 ± 0.02
	Random survival forest	0.65 ± 0.01
	Survival XGBoost	0.65 ± 0.02
NeuroBlu (external validation)	CoxPH	0.51 ± 0.01
	Random survival forest	0.56 ± 0.00
	Survival XGBoost	0.55 ± 0.00

*C*-index is reported for CoxPH, Random Survival Forest, and Survival XGBoost models.

In Table 3, which shows the time-to-event (survival) model results, Random Survival Forest showed the highest performance across both datasets, with XGBoost showing similar results on the Stanford dataset.

### Sensitivity analysis: Impact of LLM-derived clinical features and subgroup analysis

**Figure 3.**
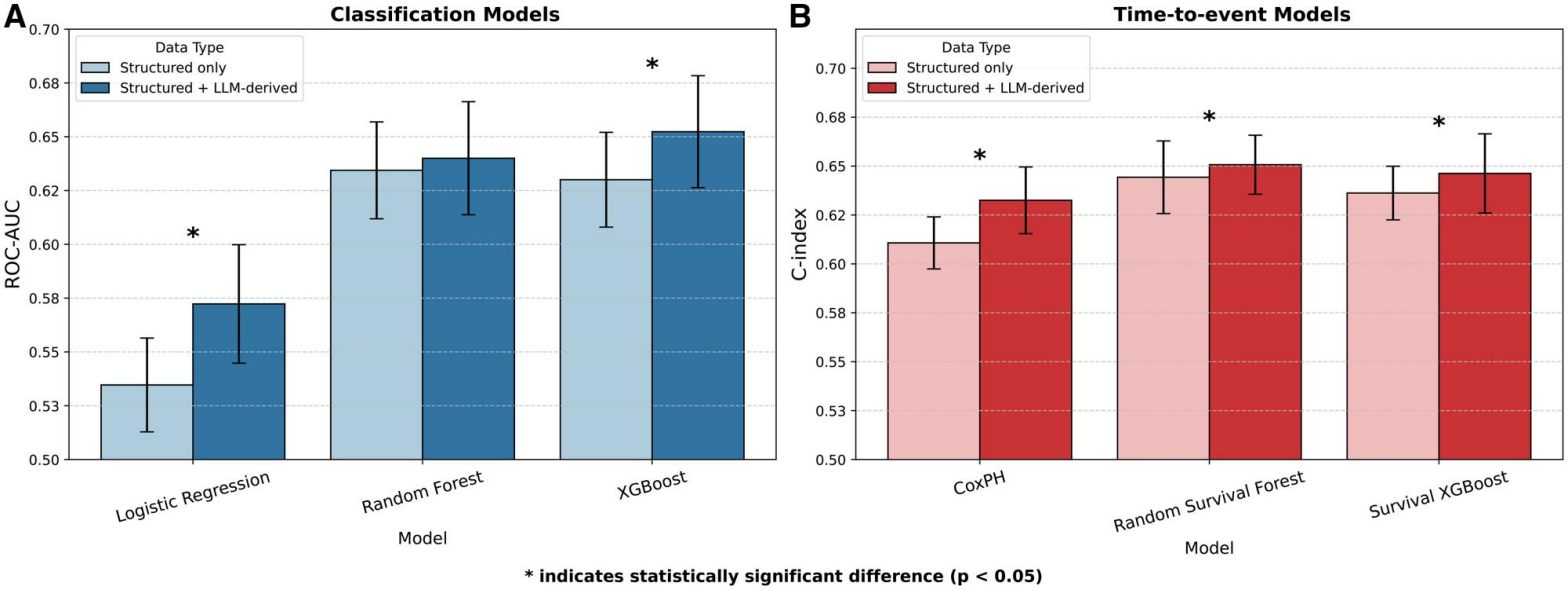
Performance comparison of classification models (A) and time-to-event models (B) using structured data alone (light colors) vs structured data augmented with LLM-derived features (dark colors). (A) ROC-AUC for classification models (Logistic Regression, Random Forest, and XGBoost), and (B) *C*-index for time-to-event models (CoxPH, Random Survival Forest, and Survival XGBoost) are shown. Error bars represent standard deviations across repeated experiments (50 iterations). Asterisks (*) indicate statistically significant differences (*P* < .05) between structured-only and LLM-augmented approaches, based on Wilcoxon signed-rank tests. Improvements were observed for the Logistic Regression, XGBoost, and all 3 time-to-event models (CoxPH, Random Survival Forest, and Survival XGBoost) when incorporating LLM-derived features.

Figure 3 illustrates the comparative performance between the models trained on structured data (193 features) and the model trained on all features, including those from unstructured data (206 features). Results demonstrate that adding LLM-derived features improved model performance across all architectures, with several models showing statistically significant improvements based on Wilcoxon signed-rank tests (*P* < .05). Among the classification models (shown in blue), Logistic Regression showed the most substantial improvement, with performance increasing from ∼0.53 to 0.57 (+4%), representing a statistically significant gain. XGBoost also demonstrated significant improvement with the addition of LLM features (>+2%), achieving the highest overall classification performance at ∼0.65. Random Forest showed modest, non-significant improvement when incorporating LLM-derived features. Time-to-event models (shown in red) consistently demonstrated modest but statistically significant improvements with the addition of LLM-derived features. CoxPH, Random Survival Forest, and Survival XGBoost all showed similar *C*-index values around 0.63-0.65 when augmented with LLM-derived features, compared to 0.61-0.64 with structured data alone. Additional statistical comparisons using Mann–Whitney U and paired t-tests were also conducted to confirm the robustness of the observed improvements. The results of these tests are provided in Table S4.

Overall, these results demonstrate that LLM-derived features can enhance model performance across different architectures, though the magnitude of improvement varies by model type and complexity. Simpler models like Logistic Regression and CoxPH showed the greatest relative improvements, while ensemble-based models such as XGBoost, Random Survival Forest, and Survival XGBoost achieved the highest absolute predictive performance.

To further evaluate the added value of LLM-derived features, we compared 4 modeling strategies. The NLP-only model (13 unstructured features) had substantially lower performance (*C*-index = 0.5364), while both combined models, whether appending or harmonizing overlapping features, outperformed the structured-only baseline (*C*-index = 0.6517), achieving similar top performance (∼0.654). These results confirm the complementary predictive value of features derived from clinical notes, even when partially redundant with structured variables (Figure S3 and Table S5).

Subgroup analysis demonstrated consistent model performance across age, gender, race, and ethnicity groups, with *C*-index values ranging from 0.60 to 0.67. Slightly lower performance was observed in the “Other race” and “Ethnicity Not Reported” categories, likely due to small sample sizes (*n* = 92 and *n* = 56, respectively) (Figure S4).

### Feature importance using SHAP

**Figure 4.**
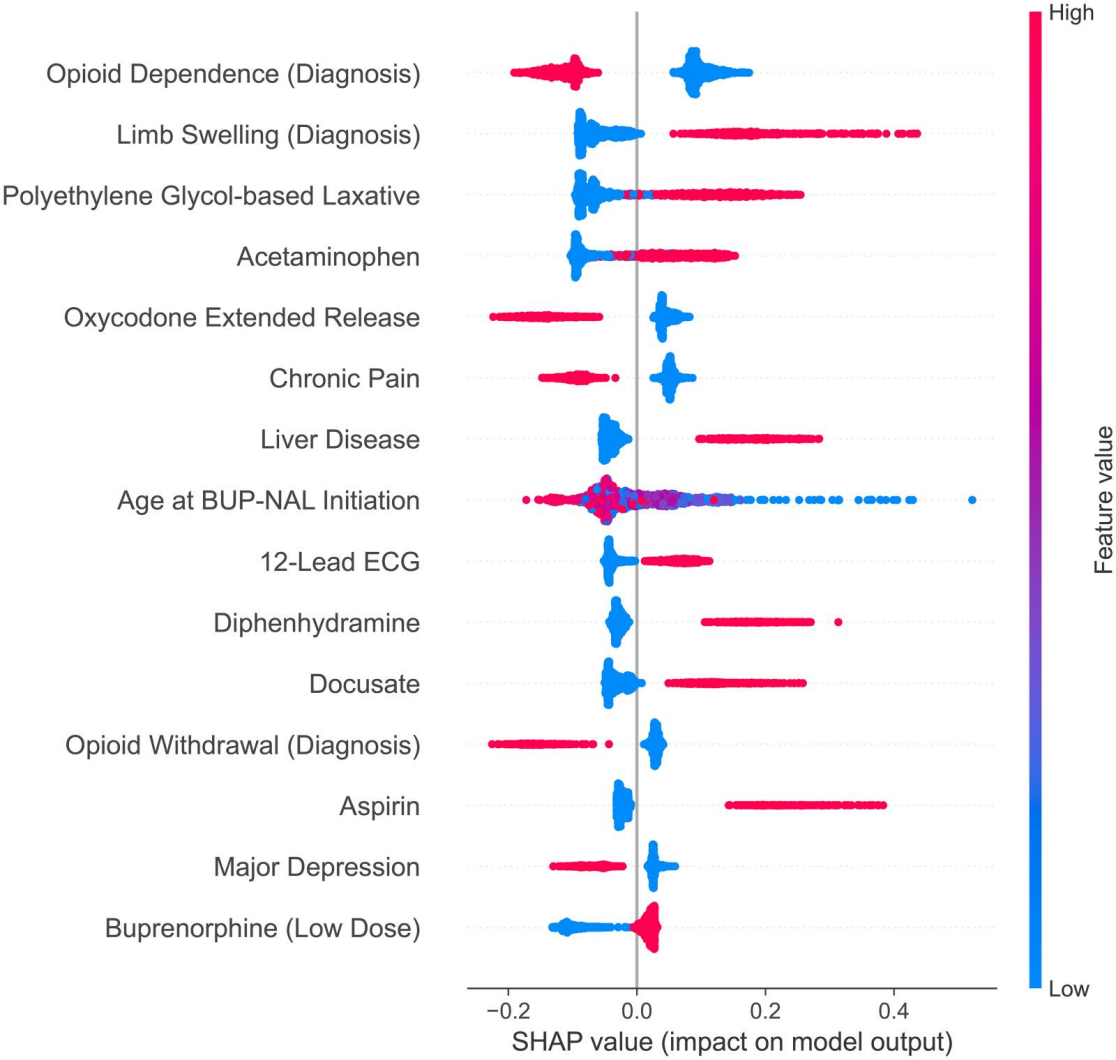
The figure shows the top 15 features ranked by their impact on the survival XGBoost model’s output for treatment attrition prediction. For example, a formal diagnosis code for Opioid Dependence was associated with a higher chance of treatment retention.

We used SHAP values to understand each feature’s contribution, ranked by their impact on the survival XGBoost model’s output for treatment attrition prediction, as illustrated in Figure 4 for the top 15 features. The SHAP summary plot depicts each feature’s impact on the model’s output along the *x*-axis, with the *y*-axis listing the features. The color of each point represents the value of the corresponding feature: for binary features, darker shades (red in Figure 4) indicate presence (coded as 1) and lighter shades (blue in Figure 4) indicate absence (coded as 0); for continuous features such as age, warmer colors correspond to higher values and cooler colors to lower values. Horizontal placement shows the extent to which each feature affects the time to attrition. Features contributing to shorter times to attrition appear to the right (positive SHAP values), while features contributing to longer times appear to the left (negative SHAP values). For example, a formally coded Opioid Dependence diagnosis (ICD-10 F11.2) is denoted by the red points and is to the left of the dividing indifference line. This means that those patients were less likely to have treatment attrition and more likely to have longer treatment retention. In contrast, if the CLEAR pipeline identified Liver Disease in a patient’s chart, that was correlated with the patient’s being more likely to have treatment attrition (and shorter treatment retention time). For a continuous valued feature (age, in this case), a higher (red) value (ie, older patient) was more correlated with treatment retention, although the spread of red and blue cases indicates that there was heterogeneity in that effect. Among the features extracted from unstructured data, Chronic Pain, Liver Disease, and Major Depression showed important contributions to the model’s predictions.

### Web application for interactive use

**Figure 5.**
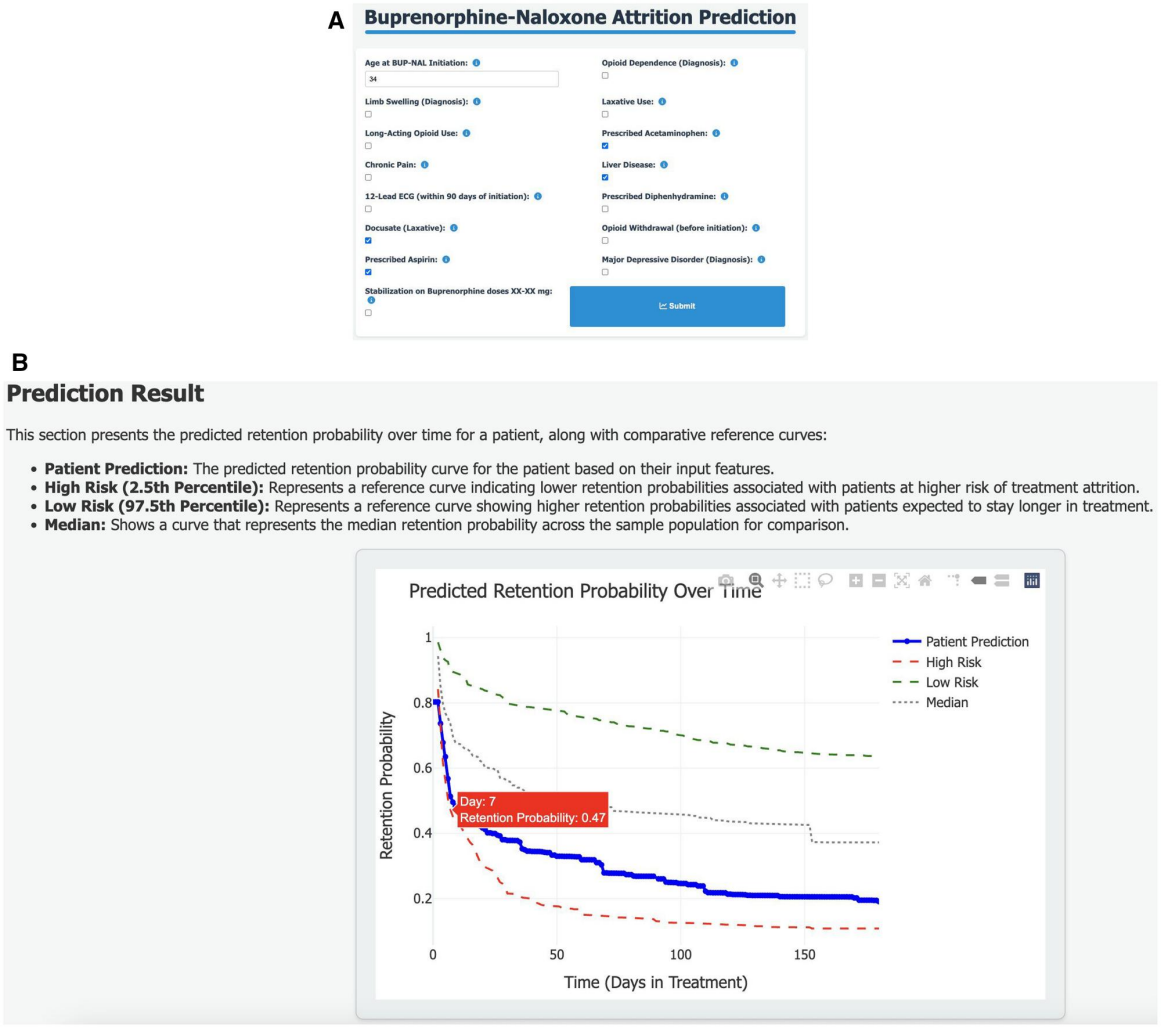
Interactive web-based tool for BUP-NAL treatment retention prediction. (A) This example features a 34-year-old patient with prescriptions for acetaminophen, aspirin, and docusate (laxative), along with a history of liver disease. (B) The personalized retention probability curve (in blue) shows a 47% likelihood of continued treatment at day 7, compared to high-risk (red), low-risk (green), and median retention (dotted gray) reference curves.

Figure 5 depicts a screenshot of an interactive web application that allows users to visualize treatment retention predictions in real time. Figure 5A shows an example for a 34-year-old patient with liver disease and prescriptions including aspirin and acetaminophen. The web application dynamically updates the patient-specific retention probability curve based on these input features. The tool provides a day-by-day probability estimate of retention up to 180 days, allowing clinicians to track the likelihood of continued treatment at any given time point. This prediction is presented together with 3 reference curves: the high-risk profile (red), which represents the 2.5th percentile of predicted retention probabilities across the population (indicating patients most likely to discontinue early); the low-risk profile (green), which represents the 97.5th percentile (patients predicted to remain in treatment the longest); and the median retention curve (dotted gray), which reflects the 50th percentile retention probability across the training sample (Stanford data). As shown in Figure 5B, the model predicts that this example patient has a 47% probability of remaining in treatment on day 7. Their predicted retention pattern matches the high-risk profile (red curve), showing a high risk of early treatment attrition. The tool is accessible at [https://www.healthrexlab.com/resources].

## Discussion

Prior research suggests that co-occurring medical and psychiatric conditions significantly influence retention in MOUD and may not be fully captured by structured data.^45^^,^^46^ In this study, we developed and evaluated multiple machine learning models to predict treatment attrition in daily oral BUP-NAL therapy using both structured EHR data and features extracted from unstructured clinical notes. We compared nonlinear machine learning models, such as Random Forest and XGBoost, with traditional statistical methods like Logistic Regression and CoxPH, given that the former can be better at capturing complex patterns in data. While these models could identify patients at risk of early treatment discontinuation, their overall discriminative power is imperfect. Our findings align with our previous research, where addiction medicine clinicians faced challenges in accurately predicting treatment retention, highlighting the complexity of this task even for experts.^18^

Across various model types, we found moderate predictive performance, with statistically significant improvements when including features from unstructured clinical notes. While it is possible that other clinical note feature extraction methods might have yielded better performance results, given the >90% agreement on the extracted features with human expert review, it is more likely that predictive performance may already be maximized for this complex biopsychosocial phenomenon that may need other data sources (or may just be fundamentally difficult to predict).

A key consideration in predictive modeling is ensuring generalizability across diverse patient populations. This study revealed significant demographic differences between the Stanford and NeuroBlu datasets, particularly in terms of age, race, and ethnic composition (Table S6). The NeuroBlu dataset represents behavioral health settings spanning hundreds of sites across the United States, encompassing a patient population with more complex behavioral health conditions and social determinants of health. This subset of NeuroBlu data reflects a higher attrition rate, likely influenced by factors such as younger age, psychiatric comorbidities, and socioeconomic barriers to treatment retention. The prevalence of features extracted from unstructured data also differed across datasets, with the NeuroBlu cohort showing higher rates of psychiatric comorbidities and social determinants of health indicators, such as homelessness, unemployment, and substance use disorders. These differences highlight the necessity of considering variations in patient populations when developing predictive models for OUD treatment retention. The contrast in attrition rates underscores the need for model validation in diverse settings to ensure reliability and external generalizability.

In this context, we made a modeling decision to restrict structured features to the intersection between Stanford and NeuroBlu datasets to ensure consistency and comparability in cross-site evaluations. While this approach may exclude informative features unique to either site, it enables a fair test of generalizability. Importantly, our prior research^19^ explored a single-site model using all locally available features, effectively capturing the predictive value of site-specific information. Thus, the current study complements that work by directly addressing the performance trade-offs of harmonization, highlighting how generalizability may be improved at the expense of local specificity. We contrast these 2 approaches to frame future directions for optimizing both performance and portability.

The identification of key clinical and psychosocial risk factors itself remains valuable, aligning with prior research on the challenges of sustaining long-term engagement in MOUD treatment.^18^^,^^47^ Notably, among the 13 features extracted from clinical notes, Chronic Pain, Liver Disease, and Major Depression were among the top 15 most significant predictors of treatment attrition out of a total of 206 features. If they were only randomly correlated, one might expect only one of the extracted text features to emerge in the top 15. Liver disease was associated with a higher likelihood of early attrition, reflecting the challenges of managing MOUD in patients with complex medical conditions. In contrast, chronic pain and major depression were associated with longer treatment retention. This could indicate increased engagement with care or closer monitoring for individuals with these comorbidities. These observations align with prior research, which indicates that psychiatric comorbidities can reduce the likelihood of patient-initiated attrition while simultaneously increasing the likelihood of treatment termination by facilities.^48^ For example, Friesen et al. found that individuals with depression, anxiety, and bipolar disorder comorbidities were less likely to initiate attrition themselves.^48^ The fact that these 3 psychiatric conditions are among the top predictors suggests that clinicians treating patients with OUD using BUP-NAL should routinely screen for these disorders, as the coexistence of medical and psychiatric conditions is critical for patient retention in MOUD.

Early identification and targeted interventions for individuals at high risk of attrition could improve long-term engagement in care. For example, high-risk individuals may be considered for early initiation of long-acting injectable and implantable formulations of buprenorphine, which have been shown to improve treatment retention and adherence by reducing the need for daily medication management.^49^ The availability of multiple extended-release versions provides additional flexibility in optimizing treatment plans based on individual patient needs.

Lower buprenorphine doses were associated with higher treatment attrition, a finding demonstrated in other recent work,^50^ reinforcing the importance of optimizing dosing in practice. Two of the features that contributed most to the attrition prediction were laxatives, which may suggest that patients already suffering constipation as a side effect of opioid use are at risk of treatment discontinuation, but it is also a treatable condition that could be intervened on early by clinicians.

Predictive modeling efforts should focus not only on improving accuracy but also on providing actionable insights that can be integrated into clinical decision support systems. To facilitate this, we developed an interactive web application that allows clinicians and researchers to explore model predictions, visualize feature importance, and assess individualized risk scores. Although machine learning models can identify patients at risk of treatment attrition, their clinical utility depends on ease of interpretation and integration into workflows. By offering an interactive platform that enhances model transparency and interpretability, the web application can bridge the gap between research and real-world decision-making. Future work should focus on validating the application in diverse clinical settings, integrating it with EHR systems, and incorporating real-time patient data to further improve its utility in guiding personalized interventions.

Several limitations in this study should be considered. While LLM-derived features were extracted using a systematic pipeline with high agreement with human expert reviewers, they may not fully capture the depth of clinical reasoning and patient narratives present in free-text notes. Aspects such as provider tone or sentiment written “between the lines,” such as expressions of concern about retention, could carry predictive value but were not explicitly incorporated in our analysis. Future research could explore sentiment analysis and NLP techniques to capture these nuanced indicators of retention risk. While our findings suggest that structured EHR data alone provides reasonable predictive power, future investigations should consider whether alternative data sources (eg, patient-reported outcomes, insurance claims, medication adherence metrics, and even genetic, wearable, and digital data trends) could yield stronger predictors of long-term MOUD engagement.

A specific limitation in interpretation is the inability of EHRs to reliably distinguish between patients prescribed BUP-NAL specifically for OUD, physiologic opioid dependence without a use disorder, or other indications, such as for pain management. Although primarily used for OUD treatment, BUP-NAL's use off-label in other situations could complicate analyses. We showed that predictive results were stable and robust even when requiring that a formal OUD diagnosis code be in the inclusion criteria, which offers.^18^ Those results indicated that BUP-NAL combination therapy (as opposed to buprenorphine or methadone monotherapy) was a better proxy for the intended study population. Another limitation is that patients may fill prescriptions outside of the healthcare systems included in this study (eg, “doctor shopping”). Though we did not find evidence of this occurring in high prevalence within our study population, this possibility could result in an underestimation of patients’ actual treatment duration, with predicted risks of attrition being worse than in reality.

While this study demonstrates the feasibility of clinical decision-support tools, additional evaluation is needed to assess the impact of model predictions on actual clinical decision-making. Prospective studies investigating whether risk stratification improves retention rates in real-world settings will be essential for refining and validating predictive approaches in OUD treatment.

## Conclusions

MOUD treatment retention is moderately predictable using structured EHR data, with some degree of transferability across clinical settings. Treatment retention is a complex and heterogeneous phenomenon that can vary due to population differences. Key risk factors can be extracted from clinical note text through advanced LLM processing methods to provide additional contextual insight. For example, mentions of Liver Disease emerge as a major predictor of treatment attrition, while mentions of Chronic Pain and Major Depression emerge as predictors of treatment retention. The ability to distinguish high vs low-risk patients supports the implementation of individualized management and targeted follow-up strategies, as well as risk adjustment, including optimization of buprenorphine dose adjustments across treatment programs. An interactive web-based tool can enhance clinical translation by allowing healthcare providers to interpret model predictions and integrate data-driven insights into treatment decisions.

## Supplementary Material

ocaf157_Supplementary_Data

## Data Availability

The data that support the findings of this study originate from Holmusk Technologies, Inc. Access to these de-identified data may be made available upon request and is subject to a license agreement with Holmusk. Contact <DataAccess@holmusk.com> to determine licensing terms. The Stanford MOUD Cohort Dataset used in this study contains identifiable protected health information and, therefore, cannot be shared publicly. Stanford University investigators, with appropriate IRB approval, can contact the authors directly regarding data access.

## Code availability

The code used to run the CLEAR NLP pipeline is available at https://github.com/iv-lop/clear. The code for the classification and time-to-event (survival) models developed in this study is available at https://github.com/HealthRex/CDSS/tree/master/scripts/OUDTreatmentRetentionVSAttrition/models
