# Supplementary material for: Predicting treatment retention in medication for opioid use disorder: a machine learning approach using NLP and LLM-derived clinical features
Source: J Am Med Inform Assoc. 2025 Sep 22;32(12):1865–76. doi: 10.1093/jamia/ocaf157 (PMC12646374; doi:10.1093/jamia/ocaf157)
Supplement: ocaf157_Supplementary_Data [file ocaf157_supplementary_data.docx]

# Supplementary Material

**Table S1:** Full list of features used in machine learning models. This table outlines the various features employed in the development of the machine learning model, grouped into categories. Each category lists specific features relevant to its classification.

| **Category** | **Features** |
| --- | --- |
| Condition | Hypokalemia, Insomnia |
| Drug | rocuronium bromide 10 MG/ML Injectable Solution, ibuprofen 600 MG Oral Tablet, thiaine hydrochloride 100 MG/ML Injectable Solution, gabapentin 600 MG Oral Tablet, docusate sodium 10 MG/ML Oral Solution, labetalol hydrochloride 5 MG/ML Injectable Solution, clindamycin 300 MG Oral Capsule, 1 ML hydralazine hydrochloride 20 MG/ML Injection, diphenhydramine hydrochloride 50 MG/ML Injectable Solution, acetaminophen 325 MG / oxycodone hydrochloride 10 MG Oral Tablet, oxycodone hydrochloride 15 MG Oral Tablet, clonazepam 0.5 MG Oral Tablet, lidocaine hydrochloride 20 MG/ML Injectable Solution, 1 ML promethazine hydrochloride 25 MG/ML Injection, haloperidol 5 MG/ML Injectable Solution, loperamide hydrochloride 2 MG Oral Capsule, bisacodyl 10 MG Rectal Suppository, tramadol hydrochloride 50 MG Oral Tablet, sennosides, USP 8.6 MG Oral Tablet, 50 ML Ceftriaxone 20 MG/ML Injectable Solution, ondansetron 8 MG Disintegrating Oral Tablet, doxycycline hyclate 100 MG Oral Capsule, calcium chloride 0.0014 MEQ/ML / potassium chloride 0.004 MEQ/ML / sodium chloride 0.103 MEQ/ML / sodium lactate 0.028 MEQ/ML Injectable Solution, 100 ML magnesium sulfate 10 MG/ML Injection, calcium chloride 0.0014 MEQ/ML / potassium chloride 0.004 MEQ/ML / sodium chloride 0.103 MEQ/ML / sodium lactate 0.028 MEQ/ML Injectable Solution, clonidine hydrochloride 0.1 MG Oral Tablet, omeprazole 20 MG Delayed Release Oral Capsule, metronidazole 500 MG Oral Tablet, glycopyrrolate 0.2 MG/ML Injectable Solution, Propofol 10 MG/ML Injectable Solution, ketorolac tromethamine 30 MG/ML Injectable Solution, alprazolam 0.5 MG Oral Tablet, buprenorphine 8 MG Sublingual Tablet, atorvastatin 20 MG Oral Tablet, 1 ML ephedrine sulfate 50 MG/ML Injection, diazepam 10 MG Oral Tablet, zolpidem tartrate 10 MG Oral Tablet, 24 HR nicotine 0.583 MG/HR Transdermal System, famotidine 10 MG/ML Injectable Solution, 2 ML prochlorperazine 5 MG/ML Injection, lorazepam 0.5 MG Oral Tablet, hydroxyzine hydrochloride 25 MG Oral Tablet, dibasic potassium phosphate 236 MG/ML / monobasic potassium phosphate 224 MG/ML Injection, polyethylene glycol 3350 17000 MG Powder for Oral Solution, phenylephrine hydrochloride 10 MG/ML Injectable Solution, midazolam 1 MG/ML Injectable Solution, melatonin 3 MG Oral Tablet, 2 ML metoclopramide 5 MG/ML Injection, dexamethasone phosphate 4 MG/ML Injectable Solution, fentanyl 0.05 MG/ML Injection, Cefazolin 2000 MG Injectable Solution, 24 HR metoprolol succinate 50 MG Extended Release Oral Tablet, calcium carbonate 1000 MG Chewable Tablet, morphine sulfate 10 MG/ML Injectable Solution, amlodipine 5 MG Oral Tablet, heparin sodium, porcine 1000 UNT/ML Injectable Solution, duloxetine 60 MG Delayed Release Oral Capsule, hydromorphone hydrochloride 10 MG/ML Injection, 0.4 ML enoxaparin sodium 100 MG/ML Prefilled Syringe, bupivacaine hydrochloride 5 MG/ML Injectable Solution, insulin, regular, human 100 UNT/ML Injectable Solution, ketamine 100 MG/ML Injectable Solution, aspirin 81 MG Delayed Release Oral Tablet, epinephrine 1 MG/ML Injectable Solution, pantoprazole 40 MG Injection, furosemide 20 MG Oral Tablet, trazodone hydrochloride 50 MG Oral Tablet, glucose 50 MG/ML / potassium chloride 0.02 MEQ/ML / sodium chloride 4.5 MG/ML Injection, magnesium hydroxide 80 MG/ML Oral Suspension, 200 ACTUAT albuterol 0.09 MG/ACTUAT Metered Dose Inhaler, naloxone hydrochloride 40 MG/ML Nasal Spray |
| Other | Depressive disorder, Acute renal failure syndrome, Tobacco dependence syndrome, Nicotine dependence, Laboratory procedure, Urinary tract infectious disease, Intravenous infusion, hydration; each additional hour (List separately in addition to code for primary procedure), Pressurized or nonpressurized inhalation treatment for acute airway obstruction for therapeutic purposes and/or for diagnostic purposes such as sputum induction with an aerosol generator, nebulizer, metered dose inhaler or intermittent positive pressure b, Therapeutic, prophylactic, or diagnostic injection (specify substance or drug); intravenous push, single or initial substance/drug, Constipation, Suicidal thoughts, Epigastric pain, Hypoxemia, Opioid abuse, Drug test(s), presumptive, any number of drug classes, any number of devices or procedures; by instrument chemistry analyzers (eg, utilizing immunoassay [eg, EIA, ELISA, EMIT, FPIA, IA, KIMS, RIA]), chromatography (eg, GC, HPLC), and mass spectrometry eit, Malaise, Therapeutic, prophylactic, or diagnostic injection (specify substance or drug); intravenous push, single or initial substance/drug, Self-care/home management training (eg, activities of daily living (ADL) and compensatory training, meal preparation, safety procedures, and instructions in use of assistive technology devices/adaptive equipment) direct one-on-one contact, each 15 minutes, Arterial catheterization or cannulation for sampling, monitoring or transfusion (separate procedure); percutaneous, Chronic pain, Cardiac arrhythmia, Preventive procedure, Backache, Major depression, single episode, Eruption, Critical care, evaluation and management of the critically ill or critically injured patient; first 30-74 minutes, Abdominal pain, Psychoactive substance abuse, Osteoarthritis, Severe recurrent major depression without psychotic features, Hypnotic or anxiolytic dependence, Alcohol dependence, Edema, Anxiety disorder, Injury of head, Traumatic or non-traumatic injury, Dyspnea, Cardiomegaly, Opioid-induced organic mental disorder, Arterial puncture, withdrawal of blood for diagnosis, Alcohol abuse, Acidosis, Atrial fibrillation, Uncomplicated asthma, Illness, Anemia, Postoperative state, Health and behavior assessment (eg, health-focused clinical interview, behavioral observations, psychophysiological monitoring, health-oriented questionnaires), each 15 minutes face-to-face with the patient; initial assessment, Self-care/home management training (eg, activities of daily living (ADL) and compensatory training, meal preparation, safety procedures, and instructions in use of assistive technology devices/adaptive equipment) direct one-on-one contact, each 15 minutes, Posttraumatic stress disorder, Pain in thoracic spine, Sepsis, Computed tomography, head or brain; without contrast material, Dehydration, Asthenia, Vitamin D deficiency, Radiologic examination, chest; single view, Obesity, Self-care/home management training (eg, activities of daily living (ADL) and compensatory training, meal preparation, safety procedures, and instructions in use of assistive technology devices/adaptive equipment) direct one-on-one contact, each 15 minutes, Nausea and vomiting, Diarrhea, Pain of left knee region, Generalized anxiety disorder, Level V - Surgical pathology, gross and microscopic examination Adrenal, resection Bone - biopsy/curettings Bone fragment(s), pathologic fracture Brain, biopsy Brain/meninges, tumor resection Breast, excision of lesion, requiring microscopic evaluation of, Chest pain, Acute posthemorrhagic anemia, Psychiatric diagnostic interview examination, Electrocardiogram abnormal, Muscle pain, Therapeutic, prophylactic, or diagnostic injection (specify substance or drug); intravenous push, single or initial substance/drug, 12 lead ECG, Essential hypertension, Health and behavior assessment (eg, health-focused clinical interview, behavioral observations, psychophysiological monitoring, health-oriented questionnaires), each 15 minutes face-to-face with the patient; initial assessment, Drug withdrawal, Anxiety state, Altered mental status, Opioid withdrawal, Gastroesophageal reflux disease without esophagitis, Low back pain, Psychological testing (includes psychodiagnostic assessment of emotionality, intellectual abilities, personality and psychopathology, eg, MMPI, Rorschach, WAIS), per hour of the psychologist's or physician's time, both face-to-face time administering test, Congestive heart failure, Disorder of soft tissue, Iron deficiency anemia, Drug abuse, Computed tomography, head or brain; without contrast material, Tachycardia, Pain, Health and behavior assessment (eg, health-focused clinical interview, behavioral observations, psychophysiological monitoring, health-oriented questionnaires), each 15 minutes face-to-face with the patient; initial assessment, Shoulder joint pain, Therapeutic procedure, 1 or more areas, each 15 minutes; gait training (includes stair climbing), Low blood pressure, Immunization administration (includes percutaneous, intradermal, subcutaneous, or intramuscular injections); 1 vaccine (single or combination vaccine/toxoid), Central venous cannula insertion, Blood chemistry abnormal, folic acid, Hypo-osmolality and or hyponatremia, Arterial catheterization or cannulation for sampling, monitoring or transfusion (separate procedure); percutaneous, Opioid dependence, Neck pain, vancomycin, Epilepsy, Type 2 diabetes mellitus without complication, Bipolar disorder, Seizure, Psychiatric diagnostic evaluation, Fever, Chronic hepatitis C, Leukocytosis |
| Treatment/Procedure | Ultrasonic guidance for needle placement (eg, biopsy, aspiration, injection, localization device), imaging supervision and interpretation, Psychotherapy, 30 minutes with patient when performed with an evaluation and management service (List separately in addition to the code for primary procedure), Intravenous infusion, for therapy, prophylaxis, or diagnosis (specify substance or drug); initial, up to 1 hour, Ultrasound guidance for vascular access requiring ultrasound evaluation of potential access sites, documentation of selected vessel patency, concurrent realtime ultrasound visualization of vascular needle entry, with permanent recording and reporting (Lis, Intravenous infusion, for therapy, prophylaxis, or diagnosis (specify substance or drug); initial, up to 1 hour, Physical therapy evaluation: moderate complexity, requiring these components: A history of present problem with 1-2 personal factors and/or comorbidities that impact the plan of care; An examination of body systems using standardized tests and measures in, Intravenous infusion, for therapy, prophylaxis, or diagnosis (specify substance or drug); initial, up to 1 hour, Physical therapy evaluation: low complexity, requiring these components: A history with no personal factors and/or comorbidities that impact the plan of care; An examination of body system(s) using standardized tests and measures addressing 1-2 elements |


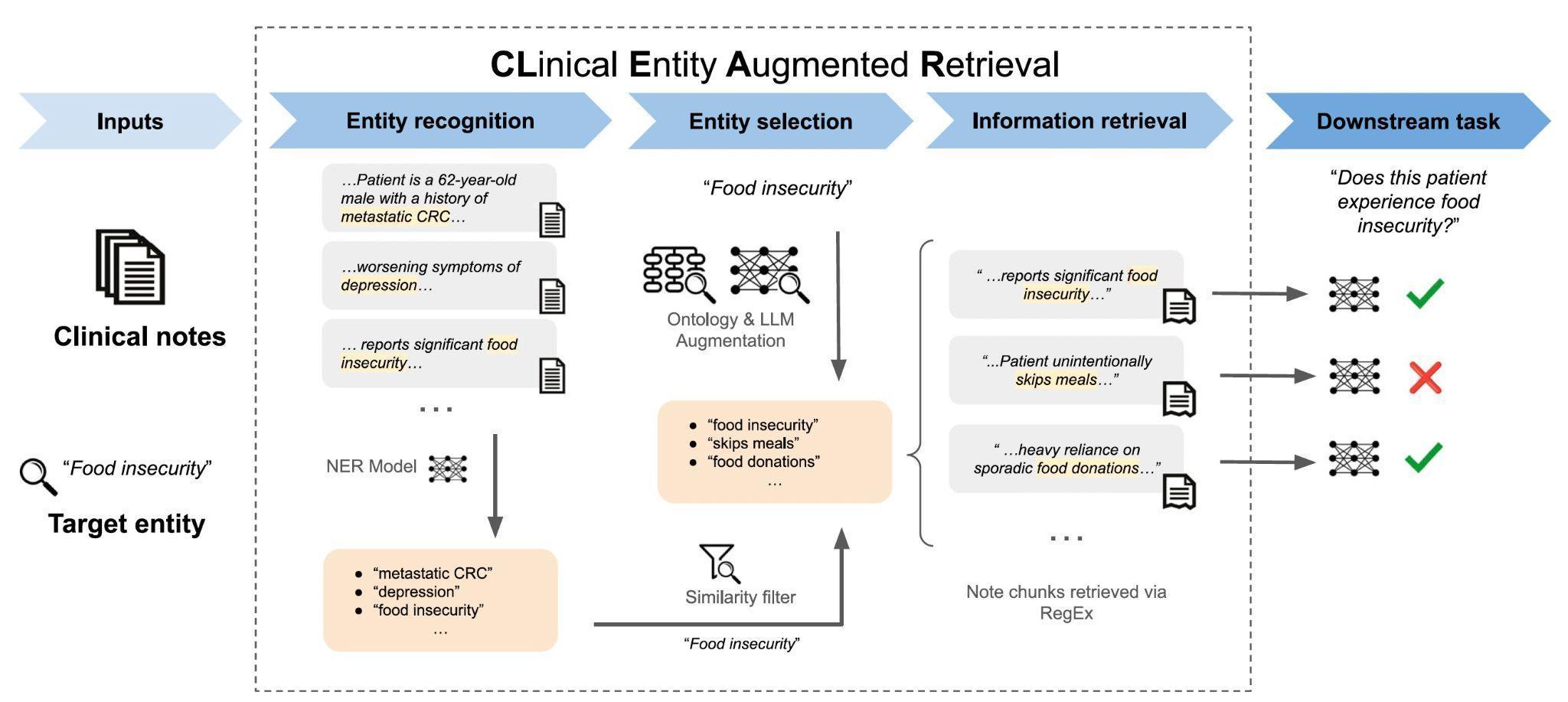


**Figure S1:** Overview of the CLEAR pipeline. CLEAR requires two inputs: (1) clinical notes and (2) a target entity. Initially, our CLEAR implementation applies an NER model to the clinical notes to extract a dataset of relevant entities. These entities are then filtered using word embeddings and cosine similarity to ensure relevance to the target entity. Next, additional entities related to the target entity are identified using ontologies and LLMs. The final list of entities is used to retrieve note chunks through regular expression matches. These chunks support a downstream LLM task (clinical information extraction).

**Table S2:** CLEAR F1 scores across six LLMs on the Stanford dataset. The final row presents the average F1 score across all variables for each model. Bolded values indicate the highest F1 score achieved per feature, highlighting the best-performing model(s) for that task. GPT-4 achieved the highest overall average (0.97), followed by Flan-UL2 (0.93) and Mixtral (0.91).

| **Feature** | **Flan-T5** | **Flan-UL2** | **GPT-4** | **Med42** | **Llama-3** | **Mixtral** |
| --- | --- | --- | --- | --- | --- | --- |
| Major Depression | 0.86 | 0.87 | **0.97** | 0.61 | 0.88 | 0.93 |
| Alcohol Dependence | 0.85 | 0.81 | **0.91** | 0.69 | 0.74 | 0.75 |
| Substance Use Disorder | 0.89 | 0.88 | **0.91** | 0.71 | 0.84 | 0.94 |
| Unhoused | 0.97 | 0.97 | 0.97 | 0.96 | **1.00** | 0.97 |
| Tobacco Dependence | 0.95 | 0.98 | **0.99** | 0.70 | 0.90 | 0.92 |
| Personality Disorder | 0.81 | 0.90 | **1.00** | 0.67 | 0.97 | 0.86 |
| Bipolar Disorder | 0.90 | 0.94 | **1.00** | 0.91 | 0.89 | 0.94 |
| PTSD | 0.95 | 0.95 | **1.00** | 0.89 | 0.96 | 0.94 |
| ADHD | 0.94 | **0.97** | **0.97** | 0.77 | 0.87 | 0.84 |
| Suicidal Behavior | 0.96 | 0.95 | **0.99** | 0.83 | 0.91 | 0.97 |
| Liver Disease | 0.82 | 0.97 | **0.99** | 0.62 | 0.81 | 0.94 |
| Chronic Pain | 0.95 | **0.97** | 0.95 | 0.88 | 0.94 | 0.94 |
| Unemployment | **1.00** | 0.98 | **1.00** | 0.88 | 0.84 | 0.95 |
| **Average** | **0.91** | **0.93** | **0.97** | **0.78** | **0.89** | **0.91** |

**Table S3:** The prevalence of the 13 LLM-derived features across the datasets.

| **Feature** | **Stanford Datatset (%)** | **NeuroBlu Dataset (%)** |
| --- | --- | --- |
| PTSD | 5.5 | 24.2 |
| Major Depression | 31.7 | 81.0 |
| Homelessness | 4.1 | 39.7 |
| Personality Disorder | 2.4 | 7.8 |
| Tobacco Dependence | 27.5 | 60.6 |
| Bipolar Disorder | 10.4 | 64.5 |
| ADHD | 6.8 | 8.5 |
| Substance Use Disorder | 40.7 | 89.8 |
| Chronic Pain | 34.3 | 31.8 |
| Suicidal Behavior | 12.7 | 79.5 |
| Unemployment | 6.2 | 42.0 |
| Alcohol Dependence | 24.3 | 80.2 |
| Liver Disease | 16.7 | 23.5 |


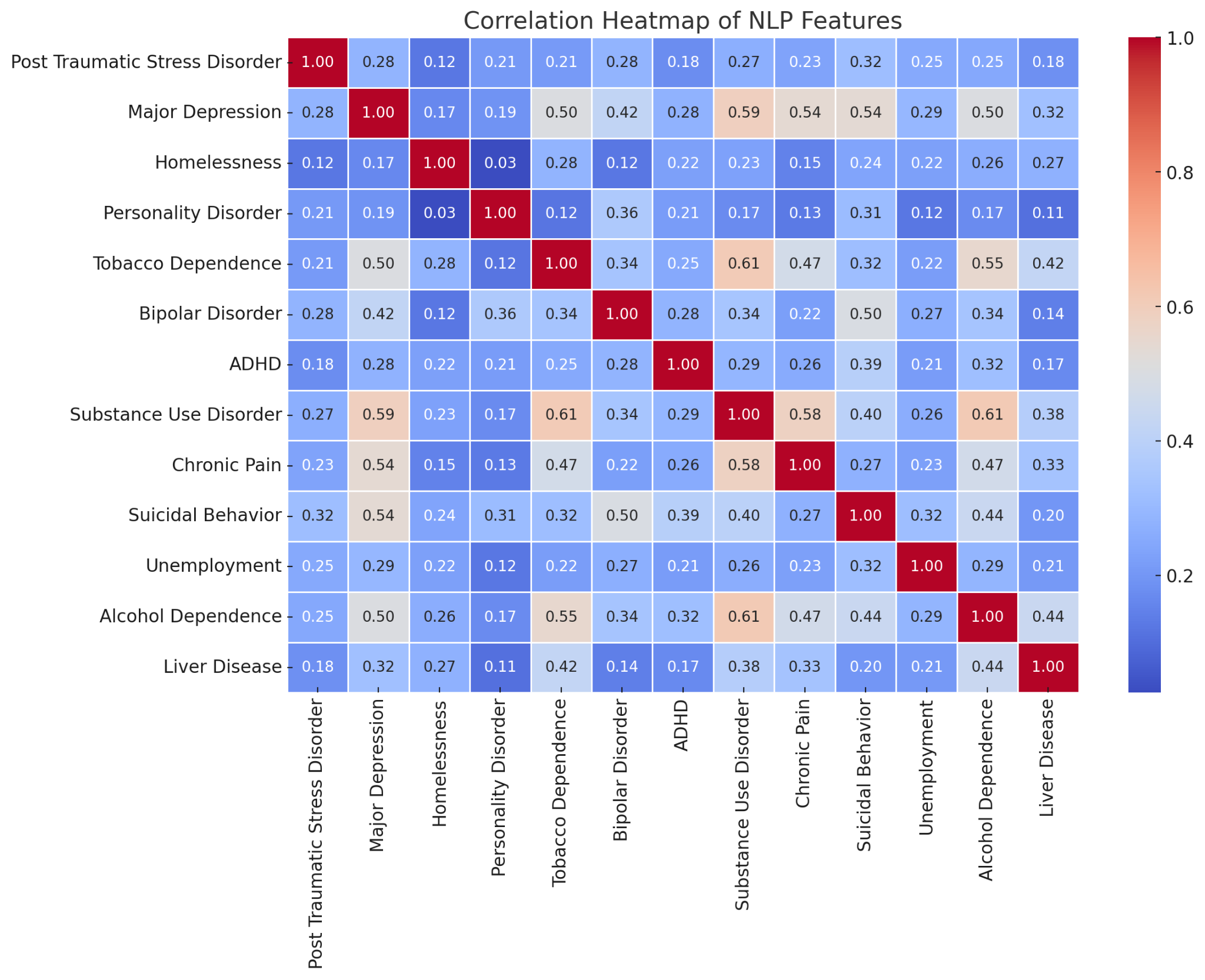


**Figure S2:** Relationships among the 13 LLM-derived features observed from Stanford data.

**Table S4:** Performance comparison of classification (ROC-AUC) and time-to-event models (C-index) using structured data alone (Baseline) versus structured data augmented with LLM-derived features (NLP). The table reports mean and standard deviation (Std) for each model under both settings, along with the absolute improvement (%) and associated p-values from three statistical tests: Wilcoxon signed-rank, Mann–Whitney U, and paired t-test.


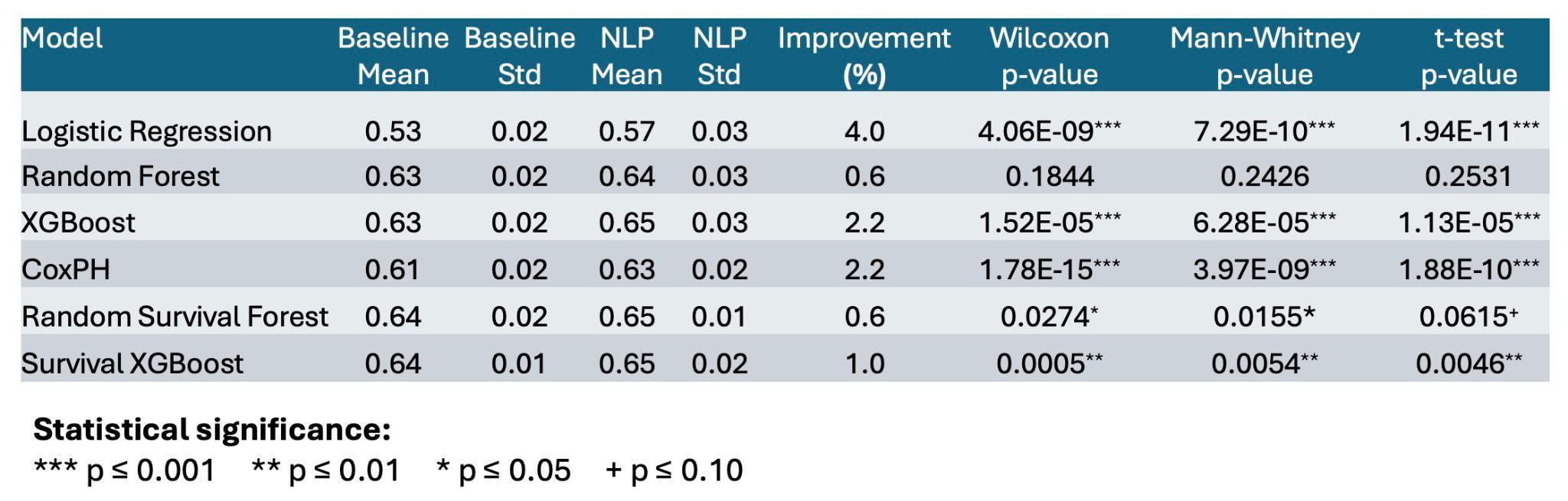


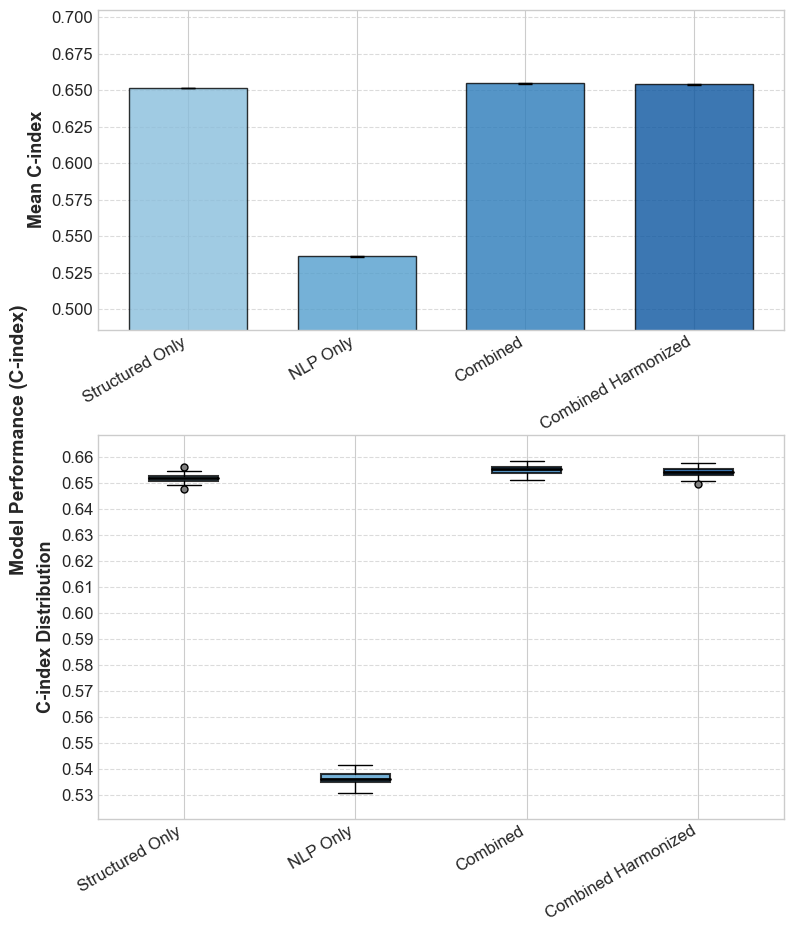


**Figure S3:** Comparison of model performance across four configurations using 100 random runs of Random Survival Forest. Top: Mean C-index with standard error bars. Bottom: Boxplot showing the distribution of C-index scores across 5-fold cross-validation.

**Table S5:** Mean C-index and standard error for each model configuration across 100 repetitions.

| **Model** | **Mean C-index** | **Standard Error** |
| --- | --- | --- |
| Structured-only | 0.6517 | 0.014 |
| NLP-only | 0.5364 | 0.022 |
| Combined | 0.6548 | 0.017 |
| Combined harmonized | 0.6541 | 0.016 |


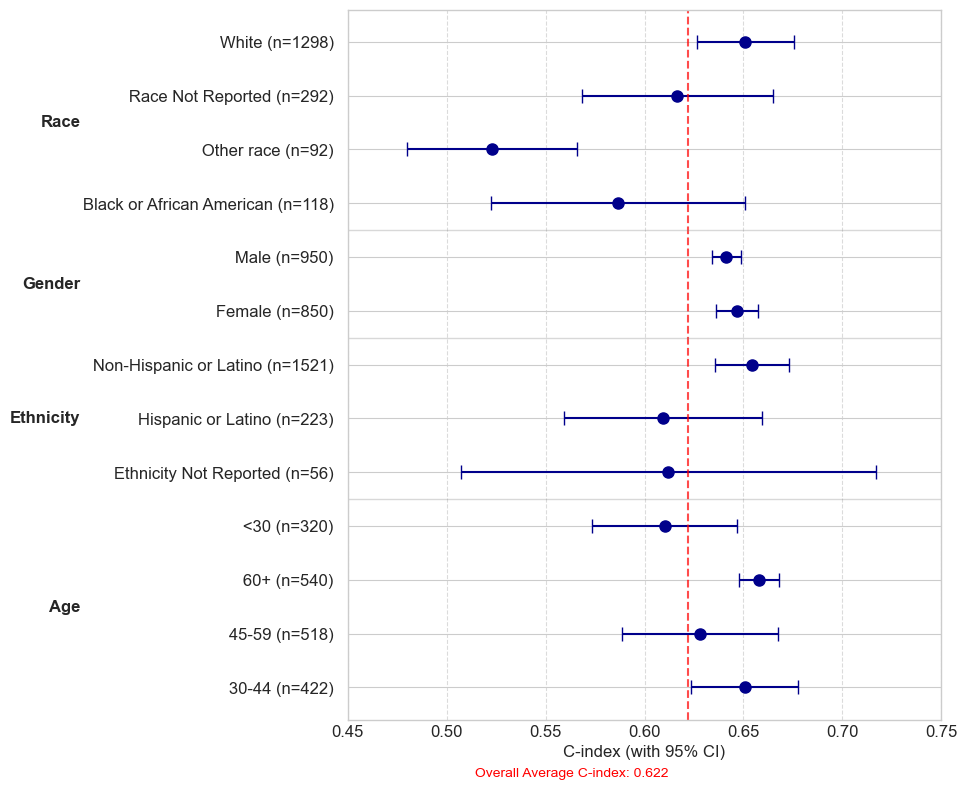


**Figure S4:** Subgroup analysis of model performance across demographic groups. C-index values (with 95% confidence intervals) are shown for subgroups stratified by age, gender, race, and ethnicity.

**Table S6:** Statistical comparison of demographic differences between Stanford and NeuroBlu Datasets.

| **Feature** | **Test Used** | **p-value** | **Statistically Significant (p < 0.05)** |
| --- | --- | --- | --- |
| Age | T-test | 1.01 × 10⁻²³ | Yes |
| Sex | Chi-Square | 0.013 | Yes |
| Race | Chi-Square | 4.55 × 10⁻¹⁴⁴ | Yes |
| Ethnicity | Chi-Square | <0.0001 | Yes |
